# Supplementary material for: Bacterial diets differentially alter lifespan and healthspan trajectories in C. elegans
Source: Commun Biol. 2020 Nov 6;3:653. doi: 10.1038/s42003-020-01379-1 (PMC7648844; doi:10.1038/s42003-020-01379-1)
Supplement: Supplementary file 2 — Description of Additional Supplementary Files [file 42003_2020_1379_MOESM2_ESM.pdf]

Description of Additional Supplementary Files

File Name: Supplementary Data 1

Description: RNAseq analysis in L4 C. elegans.

File Name: Supplementary Data 2

Description: Lifespan quartile comparisons.

File Name: Supplementary Data 3

Description: Thrashing replicates at each life stage.

File Name: Supplementary Data 4

Description: Bacterial diet as a nutraceutical Log-rank analysis compared to OP50, Red, and Orange.

File Name: Supplementary Data 5

Description: Graph and chart source data in all main figures.
